# Alzheimer-associated cerebrospinal fluid fragments of neurogranin are generated by Calpain-1 and prolyl endopeptidase

**DOI:** 10.1186/s13024-018-0279-z

**Published:** 2018-08-29

**Authors:** Bruno Becker, Faisal Hayat Nazir, Gunnar Brinkmalm, Elena Camporesi, Hlin Kvartsberg, Erik Portelius, Martina Boström, Marie Kalm, Kina Höglund, Maria Olsson, Henrik Zetterberg, Kaj Blennow

**Affiliations:** 1 Department of Psychiatry and Neurochemistry, Institute of Neuroscience and Physiology, The Sahlgrenska Academy at University of Gothenburg, Gothenburg, Sweden; 2 Clinical Neurochemistry Laboratory, Sahlgrenska University Hospital, Mölndal, Sweden; 3 Department of Pharmacology, Institute of Neuroscience and Physiology, The Sahlgrenska Academy at University of Gothenburg, Gothenburg, Sweden; 4 Department of Molecular Neuroscience, UCL Institute of Neurology, University College London, Queen Square, London, UK; 5 UK Dementia Research Institute at UCL, London, UK

**Keywords:** Neurogranin, Calpain, Prolyl endopeptidase, Postsynaptic protein, IQ domain, Biomarker, Alzheimer’s disease

## Abstract

**Background:**

Neurogranin (Ng) is a small 7.6 kDa postsynaptic protein that has been detected at elevated concentrations in cerebrospinal fluid (CSF) of patients with Alzheimer’s disease (AD), both as a full-length molecule and as fragments from its C-terminal half. Ng is involved in postsynaptic calcium (Ca) signal transduction and memory formation via binding to calmodulin in a Ca-dependent manner. The mechanism of Ng secretion from neurons to CSF is currently unknown, but enzymatic cleavage of Ng may be of relevance. Therefore, the aim of the study was to identify the enzymes responsible for the cleavage of Ng, yielding the Ng fragment pattern of C-terminal fragments detectable and increased in CSF of AD patients.

**Methods:**

Fluorigenic quenched FRET probes containing sequences of Ng were utilized to identify Ng cleaving activities among enzymes known to have increased activity in AD and in chromatographically fractionated mouse brain extracts.

**Results:**

Human Calpain-1 and prolyl endopeptidase were identified as the candidate enzymes involved in the formation of endogenous Ng peptides present in CSF, cleaving mainly in the central region of Ng, and between amino acids 75_76 in the Ng sequence, respectively. The cleavage by Calpain-1 affects the IQ domain of Ng, which may deactivate or change the function of Ng in Ca^2+^/calmodulin -dependent signaling for synaptic plasticity. While shorter Ng fragments were readily cleaved in vitro by prolyl endopeptidase, the efficiency of cleavage on larger Ng fragments was much lower.

**Conclusions:**

Calpain-1 and prolyl endopeptidase cleave Ng in the IQ domain and near the C-terminus, respectively, yielding specific fragments of Ng in CSF. These fragments may give clues to the roles of increased activities of these enzymes in the pathophysiology of AD, and provide possible targets for pharmacologic intervention.

**Electronic supplementary material:**

The online version of this article (10.1186/s13024-018-0279-z) contains supplementary material, which is available to authorized users.

## Background

Synapses are the key physiological units in the central nervous system (CNS) and are central for neuronal signal transmission as well as memory and cognitive functions. Disturbances in memory formation, as observed in Alzheimer’s disease (AD), are associated with synaptic degeneration and loss [1]. Indeed, a decreased density of synapses in mid-frontal and inferior parietal sections of the brain has been found to show tighter correlation to cognitive impairment in AD than amyloid plaques and tangles [2]. The measurement of synaptic proteins in cerebrospinal fluid (CSF), released during synaptic degeneration, is therefore a possible route to monitor synaptic pathophysiology in man [3]. Further, the mechanism of release of synaptic proteins may involve one of several conventional or non-conventional active mechanisms [4], or merely reflect passive secretion occurring during cell death which may be more relevant at later stages of AD.

Recent research showed that particular synaptic proteins, such as neurogranin (Ng) can be detected in CSF at increased levels already at early stages of AD, both as apparently full length protein [5] and as proteolytic endogenous fragments [6].

Increased concentrations of Ng in CSF correlate with future rate of cognitive deterioration and other measures of disease progression [6, 7], and the increase appears to be specific to AD and is not seen in many other neurodegenerative diseases [8, 9].

Ng is a 7.6 kDa neuronal protein [10–13] localized primarily in the cell body and dendrites. By using immunogold electron microscopy, Ng has been detected preferentially near the plasma membrane of dendritic spines [14]. Ng has two known mutually exclusive intracellular binding partners, calmodulin (CaM) [15] and phosphatidic acid (PA) [16] . Essential for this binding is the IQ domain of Ng, so-called because of the presence of the amino acids isoleucine (I) and glutamine (Q). The IQ domain is well conserved among a number of CaM-binding neuronal proteins, such as growth-associated protein 43 (GAP-43) and PEP-19 (also called purkinje cell protein 4, pcp4) [17]. Moreover, the main function for Ng appears to be to modulate CaM’s signal transduction pathways of CaM dependent enzymes to enhance synaptic plasticity [12] in long term potentiation (LTP).

Molecular characterization of Ng in the CSF by immuno-enrichment using a specific Ng antibody and subsequent immunoblotting suggested that Ng is present as a protein with an apparent molecular weight of ~ 12 kDa [5]. However, detection by mass spectrometry showed various endogenous fragments representing approximately the C-terminal half, all but one lacking the IQ domain [6]. Fragments representing the N-terminal half were not identified in that study. The C-terminal fragments are not generated by in vitro degradation of the protein in the CSF [18] and may instead be released from degenerating synapses in the CNS during the progression of the disease. These C-terminal Ng fragments were frequently truncated at Ng75, thus missing the last three amino acids (-SGD) at the C-terminus. Altogether, it appears that in CSF, Ng is present both as a full-length molecule and as fragments where major cleavages seem to be in the middle region and at/or near position 75. The enzyme(s) responsible for the generation of those fragments of Ng are unknown.

The important role of Ng in synaptic function and cognition was highlighted from a gene deletion mouse model that illustrated the deficits of these animals in learning and memory. Ng deficient mice also exhibited several behavioral abnormalities, most severe in the Ng knock-out mice (−/−), and less severe in hemizygote mice (−/+) with intermediate expression of Ng [19]. On the other hand, overexpression of Ng seems to be able to counteract negative effects of amyloid β (Aβ) oligomers on synaptic transmission, as determined by measuring evoked excitatory postsynaptic currents (EPSC) on Ng overexpressing neurons in rat hippocampal slices [20]. Thus, it appears that maintenance of a normal level of this protein in neurons is essential for proper cognition and a reduction of Ng upon aging and degenerative diseases may result in negative cognitive symptoms.

Enzymatic cleavage of Ng may be the first step towards a diminished function of Ng, or its loss to the brain interstitial fluid and, consequently, CSF. The aim of this study was to identify enzymes that can produce Ng fragments having the same or similar N- and C-termini as seen on some of the most prominent C-terminal peptides identified in the CSF and brain of patients with AD.

## Methods

### Animals

Female C57BL/6 J mice from Charles River Laboratories (Sulzfeld, Germany) were used in this study. The mice were kept on a 12-h light cycle with food and water provided ad libitum*.* The room temperature was 19–21 °C with 40–70% relative humidity. All experiments were approved by the Swedish Animal Welfare Agency.

### Preparation of mouse brain extract

The brains from ten eight-week-old female mice were quickly removed after sacrifice, put in liquid nitrogen, and stored at − 80 °C. While still frozen, the cerebelli were pinched off with a spatula. Homogenization of the brain tissue (3.48 g) was performed with 3 to 4 bursts of about 30 s with an Ultra-Turrax homogenizer (IKA T10 basic) with disposable tips (S10D-7G-KS-65) on ice. The homogenization buffer (50 mM Tris-HCl, pH 7.8, 1 mM DTT; ice cold) was used in about six times excess (21 mL) over the total wet brain weights. Homogenization was finished within 10 min. The raw extract was then diluted with ice-cold homogenization buffer to 35 mL and centrifuged 30 min at + 4 °C at 40,000 x *g* (SW28 rotor on Beckman ultracentrifuge). Further, the supernatant was centrifuged again at 100,000 x *g*, + 4 °C for one hour, aliquoted and stored at − 80 °C (protein concentration by BioRad DC protein test: 3.3 mg/mL; calibrator: BSA).

### Antibodies

The C-terminal region specific neurogranin antibodies NG36 and NG22 were prepared in-house using human peptides Ng63–77 and Ng63-76, respectively, as immunogens, and the neurogranin antibody H-6 was purchased from Santa Cruz Biotechnologies (#sc-514922).

### Gel electrophoresis and western-blotting

SDS PAGE gels (12% BisTris) were run in MES buffer and equilibrated afterwards for 25 min in 1× transfer buffer containing 20% MeOH (all gels and buffers from BioRad or Life Technologies). The proteins were transferred to a nitrocellulose membrane (Protran 0.2 μm; Amersham #10600001) in a semi-dry blot apparatus at 0.8 mA/cm^2^ at room temperature for 60 min, followed by drying of the membrane overnight. Further fixing of the transferred proteins was done by incubating the membrane in 0.4% formaldehyde (1:40 dilution of Thermo Sci # 28906) in PBS for 30 min at room temperature under light shaking. After three washes (10 min) with PBST (PBS with 0.05% Tween 20), the membrane was blocked with 5% dry milk powder (BioRad #170–6404) in PBST. Further processing and detection of signals by ECL (GE Healthcare, #RPN2235) was done according to standard procedures.

### Detection of cleavage of quenched FRET probes by calpain-1

The quenched FRET peptides ((2-Abz)-AKIQASFRGHK(Dnp), (2-Abz)-GHMARKKIKSGERK(Dnp), and (2-Abz)-GAGGGPSGDK(Dnp) (Caslo, Denmark) containing sequences Ng31–40, Ng39–51, and Ng70–78, respectively, were each digested with calpain-1 at room temperature. The assay mix (80 μL per well in a 384-well plate; AlphaPlate-384, PerkinElmer) consisted of 12.5 μM quenched peptide in 25 mM Tris-HCl, pH 7.5, 1 mM CaCl_2_ and 6 units of calpain-1 (hu erythrocytes calpain-1; Millipore 208713). Controls contained assay buffer and quenched peptide but no enzyme. The developing fluorescence was measured during 30 min on a Molecular Devices Spectramax Gemini reader (excitation 320 nm, emission 420 nm).

### Preparation of untagged recombinant Ng via 6xHis-SUMO-Ng fusion protein

Human Ng1–78 cDNA was PCR-amplified using the ORF clone RC201209 from Origene as template and primers 5’ATGGACTGCTGCACCGAGAAC 3′ and 5´ CTAGTCTCCGCTGGGGCCGCCGCC 3′. Cloning into the pET_SUMO vector and protein expression in *E. coli* BL21 (DE3) were performed according instructions of the “Champion pET SUMO expression system” (Invitrogen #K30001). The expressed 6xHis-SUMO-Ng fusion protein in the *E. coli* cell pellet was extracted with 5 mL/mg 1× Bind/Wash buffer (50 mM sodium-phosphate, pH 8.0, 300 mM NaCl, 0.01% Tween 20) plus 0.5% NP40 and incubated with rotation at room temperature for 30 min, after which the lysate was centrifuged at 17000 x *g* for 20 min at + 4 °C and the supernatant was collected. Further, the fusion protein was isolated using the “Dynabeads His-tag Isolation & Pulldown” kit (Life Technologies # 10104D). The fusion protein (approximately 400 μg from 1 L initial culture) was concentrated and the buffer exchanged to 20 mM Tris-HCl, pH 8.0, 150 mM NaCl, 1 mM DTT on Amicon Ultra 4 (Merck Millipore) with molecular weight cut-off (MWCO) 10 K. Cleavage reaction to remove the 6xHis-SUMO tag was performed according to instructions using materials of the pET SUMO expression system kit. Briefly, a 200 μL digestion mix contained 20 μg fusion protein, 1× SUMO Protease buffer without salt, and SUMO Protease 10 μL (10 U). Digestion was carried out at + 30 °C for four hours. Removal of 6xHis-SUMO tag and His-tagged SUMO Protease was done with the “Dynabeads His-tag Isolation & Pulldown” kit. Beads were collected and the untagged protein in the supernatant was concentrated and the buffer was exchanged to 20 mM HEPES, 300 mM NaCl, 2 mM TCEP, pH 7.5 on Amicon Ultra 4, 3 k MWCO (molecular weight cut-off).

### In vitro cleavage of Ng-Myc-DDK fusion protein by calpain-1

Recombinant Ng-Myc-DDK fusion protein (1.6 μg in 20 μL; Origene Technologies TP301209) was incubated in the presence of 100 μM CaCl_2_ with 2 μL calpain-1 dilution in a final volume of 40 μL for 1 h at room temperature. The calpain-1 dilutions were prepared by diluting the enzyme stock solution (human erythrocytes calpain-1; Millipore #208713; 1.68 mg/mL; specific activity 2069 units/mg) in 25 mM Tris-HCl, pH 7.5, in various dilutions (3.3 to 90-fold). Aliquots of half (20 μL) of the cleavage mixtures were used for SDS/PAGE (reducing conditions) and the remaining halves (20 μL) for mass spectrometric analysis (described further below).

### In vitro cleavage of recombinant Ng1–78 protein by calpain-1

Approximately 1.5 μg (7.5 μL) recombinant untagged Ng were digested in the presence of 100 μM calcium chloride and 12 mM Tris-HCl, pH 7.5 with 1 μL calpain-1 dilution in a total volume of 20 μL. Calpain enzyme was the same as used for the cleavage of Ng-Myc-DDK fusion proteins, at 10-, 30- and 90-fold dilutions in 25 mM Tris-HCl, pH 7.5. The digestions were carried out for 90 min at room temperature. Most of each sample (15 μL) was used for SDS-PAGE (12%) followed by Coomassie and silver staining, and 2.5 μL for each of the immunoblots (NG36 and H-6 antibodies).

### Mass spectrometric analysis of calpain-1 cleavage products

Twenty μL of the calpain-1 digests of Ng-Myc-DDK fusion protein were acidified with 1.6 μL 10% trifluoroacetic acid and frozen before the analysis. Nanoflow liquid chromatography (LC) coupled to electrospray ionization (ESI) high resolution hybrid quadrupole–orbitrap mass spectrometry (MS) was performed with a Dionex 3000 system and a Q Exactive (both Thermo Fisher Scientific, Inc.). The centrifuged sample solution was loaded onto an Acclaim PepMap C18 trap column (length 20 mm, i.d. 75 μm, particle size 3 μm, pore size 100 Å, Thermo Fisher Scientific, Inc.) for desalting and sample cleanup. Sample loading buffer was 0.05% trifluoroacetic acid/2% acetonitrile/water (v/v/v). Separation was performed by a reversed-phase Acclaim PepMap C18 analytical column (length 150 mm, i.d. 75 μm, particle size 2 μm, pore size 100 Å, Thermo Fisher Scientific, Inc.). Separation was performed at a flow rate of 300 nL/min by applying a 50 min long linear gradient from 3 to 40% B. Buffer A was 0.1% formic acid/water (v/v) and buffer B was 0.1% formic acid/84% acetonitrile/water (v/v/v). The mass spectrometer was set to operate in data-dependent mode, i.e., acquiring fragment mass spectra whenever the acquisition software detected peptide peaks above threshold intensity. Spectrum deconvolution and database searches were performed either using Mascot Distiller 2.6.3 and Mascot Daemon 2.6.0/Mascot search engine 2.6.1 or PEAKS Studio 8.5 against a custom-made neurogranin database as well as Uniprot KB human.

### Size exclusion chromatography (SEC) of preparations with Ng C-terminal cleaving activity

An aliquot (500 μL) of sample (raw or partially purified brain extract) was injected onto a 300 × 10 mm Superdex 200 column (GE Healthcare) in a cold room (+ 4 °C). Separation was performed at 0.4 mL/min and fractions of 0.5 mL were collected. Prior to the separation, the column had been calibrated using blue dextran (marking void volume), bovine serum albumin (66 kDa), carbonic anhydrase (29 kDa; from bovine erythrocytes), cytochrome C (12.4 kDa; from horse heart) and aprotinin (6.5 kDa; from bovine lung) as size markers. C-terminal cleaving activity was determined in the collected fractions using the fluorogenic assay described below.

### Fluorogenic assay for detecting C-terminal cleavage activity

The assay utilized the quenched fluorogenic peptide Ng70–78 ((2-Abz)-GAGGGPSGDK(Dnp)) at 12.5 μM and 1 mM CaCl_2_. CaCl_2_ in the assay buffer should enable activity of Ca^2+^-dependent proteases, potentially present in the samples. In the assay, samples (52 μL; e.g. ion-exchange or size exclusion chromatography (SEC) column fractions) were combined with 28 μL of a mix of 220 μL 50 μM quenched peptide and 88 μL 10 mM CaCl_2_. Fluorescence increase was recorded in kinetic mode on a Spectramax Gemini at 320 nm/420 nm (excitation/emission) in wells of a 384-well plate (PerkinElmer, #FP1342, black) for up to 30 min at room temperature.

### Chromatographic enrichment of enzymatic activity from mouse brain extract

A Tricorn 10/100 mm column (GE Healthcare) was filled with Q-Sepharose high performance gel (GE 17–1014-01) and equilibrated in a cold room (+ 4 °C) in buffer A (25 mM Tris-HCl pH 7.5, 10% glycerol). Mouse brain extract (10 mL; 3.3 mg protein/mL) was injected into the column and separation was performed using a linear gradient of buffer A and B (0–20 min: 0% B; 20–60 min: 0–50% B; 60–63 min: 50–80% B; 63–68 min: 80% B; 68–71 min: 80–0% B; 71–91 min: 0% B) at a flow rate of 1 mL/min. Buffer B was buffer A with added NaCl (to 1 M). Fractions of 1 mL were collected and aliquots analyzed for enzymatic activity.

Active fractions #40–45 from the Q-Sepharose column were pooled (6 mL) and concentrated on YM-10 ultrafiltration membranes (Centricon tubes, 2 mL; #4205, Amicon). An aliquot (approximately 2.2 mL) of the active pool was applied to the device and spun at + 4 °C, 5000 x *g* for about 80 min in a SM-24 rotor (Sorvall) to yield a concentrate of about 800 μL. The tube was refilled with more of the active pool and concentrated again; this was repeated four more times until about 1 mL of concentrate and 5 mL of filtrate were obtained. Total time of centrifugation was approximately 5.5 h. Prior to an additional anion-exchange separation on a high resolving Mono Q column, the concentrate was desalted by SEC (Superdex 200 column, as described above). Fractions containing C-terminal cleaving activity on neurogranin (#23–28) were pooled and stored at + 4 °C prior to the Mono Q column separation step.

The pool of active fractions from the Superdex 200 column (approx. 2.5 mL) was diluted with the same volume of 10% glycerol to reduce the salt concentration to 25 mM. In a cold room (+ 4 °C), this sample was applied onto a pre-equilibrated Mono Q column (5 × 50 mm; Pharmacia #17–0546-01) and eluted with a linear gradient of buffer A (25 mM Tris-HCl pH 7.5, 10% glycerol) and B (=A with 1 M NaCl) at a flow rate of 0.5 mL/min. Gradient profile: 0–10.5 min: 0% B; 10.5–40.5 min: 0–40% B; 40.5–45.5 min: 40–80% B; 45.5–50.5 min: 80% B; 50.5–53.5 min: 80–0% B. During loading and before start of the gradient, ten fractions of 1 mL were collected, thereafter the fraction size was reduced to 0.5 mL. The fractions were analyzed for enzymatic activity and stored at + 4 °C before the next step of analysis (native gel overlay).

### Native gel overlay with fluorogenic substrate

Prior to native gel electrophoresis, 150 μL of the fraction with peak enzymatic activity from the Mono Q column (fraction 38) was further concentrated and desalted in a 0.5 mL 10 K spin filter (Millipore UFC501024) at 14,000 *x g*, + 4 °C, 30 min. Briefly, the concentrate (50 μL) was diluted with 150 μL cold 25 mM Tris-HCl, pH 7.5, 10% glycerol, and concentrated again as before. The dilution and concentration was repeated once more. Fifteen μL of the final concentrate was mixed with 15 μL native gel sample buffer (2×; Novex LC2673) and loaded on a 10% Tris-Glycine gel (Lifetechnologies EC6075BOX) using Novex Tris-Glycine native gel running buffer. After electrophoresis (110 V, 2.25 h), the gel was briefly rinsed in water and incubated in 50 mM Tris-HCl pH 7.5 for 10 min with light shaking. Further, the gel was soaked in 5 μM fluorescence-quenched peptide 5-FAM-GAGGGPSGDK(QXL520) (Eurogentec) in 50 mM Tris-HCl, pH 7.5 for 15 min before imaging on a UV bench using a FITC filter set on the LAS3000 imager (Fujibio). The UV bench had an additional “Superbright” filter (Vilber Lourmat) to reduce background of non-UV light. Fluorescent bands and non-fluorescent control bands were cut out from the gel and processed for mass spectrometric analysis.

### In vitro cleavage of Ng1–78 and Ng43–78 by human PREP

The cleavage reaction contained 400 ng of Ng peptide, 20 mM NaCl, 25 mM Tris-HCl, pH 7.5, and 1 μL of recombinant human PREP (R&D Systems, #4308-SE-010; 0.44 mg/mL) in a total volume of 10 μL. After one and four hours at 37 °C, samples of 5 μL were taken, diluted on ice with 5 μL water and directly analyzed by LC-MS/MS.

### Mass spectrometric identification of C-terminally cleaving enzyme

The cut out bands were processed according to a standard protocol (reduction with DTT, followed by alkylation of reactive cysteines with iodoacetamide and digestion with trypsin). LC-MS/MS analysis was performed as described above except that an Orbitrap Fusion mass spectrometer (Thermo Fisher Scientific, Inc.) was used instead of a Q Exactive. Peptides were searched using Mascot search engine 2.5.1 against Swiss-Prot database (taxonomy: *Mus musculus*).

## Results

### Identification of calpain-1 as the enzyme cleaving Ng in the IQ domain

**Fig. 1:**
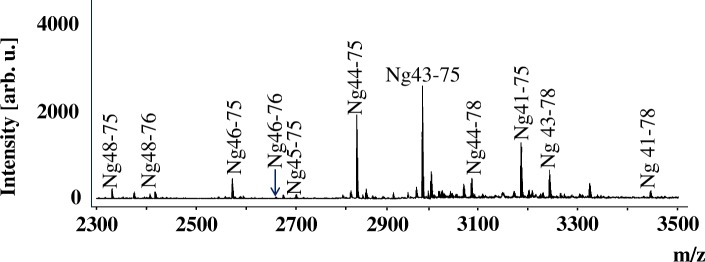
Representative hybrid immunoaffinity (HI)-MS spectrum of Ng CSF peptides identified in CSF

**Fig. 2:**
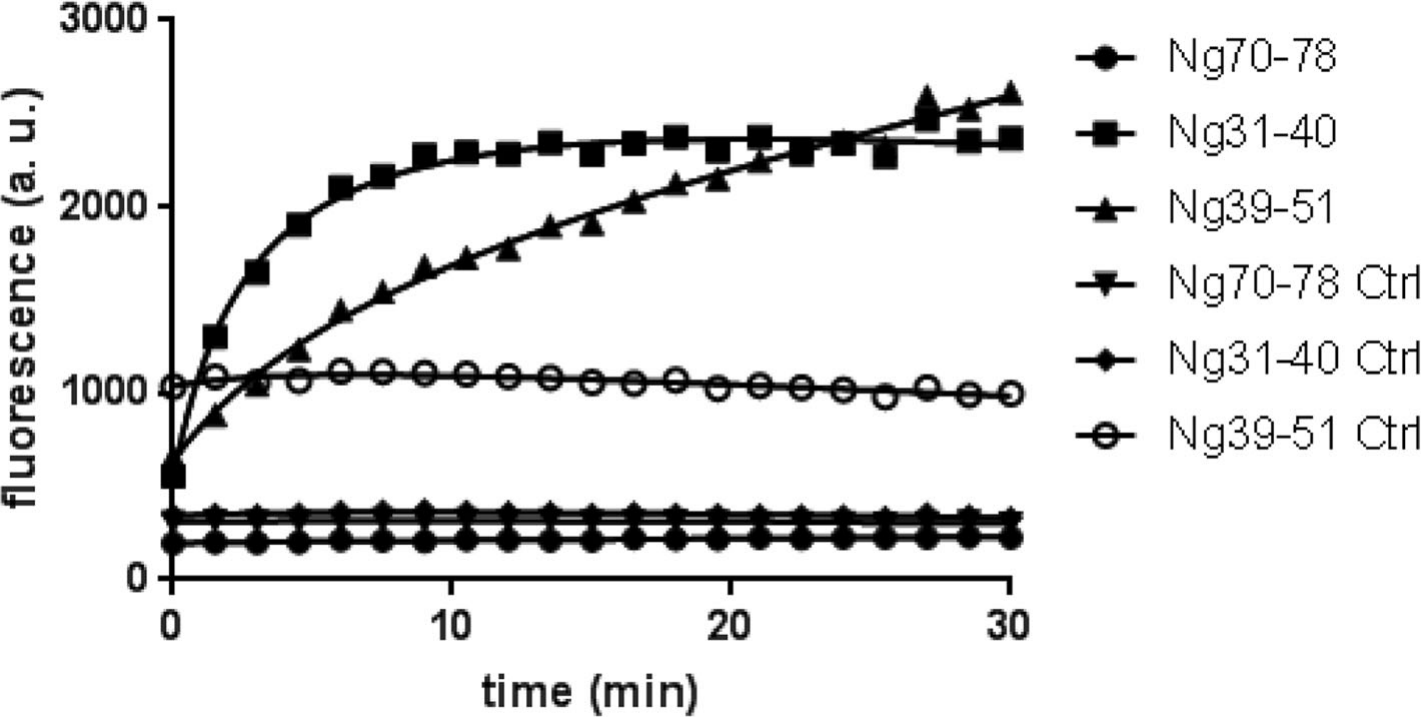
Calpain-1 cleaves the two quenched Ng peptides Ng39–51 and Ng31–40 (central region) but not the quenched peptide Ng70–78 (C-terminal region). Ng39–51 Ctrl, Ng31–40 Ctrl, Ng70–78 Ctrl, quenched peptide in assay buffer without calpain I

Most of the Ng peptides identified in human CSF showed N-terminal endings between amino acid (aa) 41 and 49 and C-terminal endings between aa 75 and 78 (see Fig. 1; modified from ref. [6]). Hence, fluorescence-quenched FRET probes encompassing these sequence regions were utilized to detect relevant endogenous cleaving activity for Ng in brain tissue, the likely source for the CNS Ng peptides. It has been reported that excitatory overstimulation of neurons can lead to excitotoxicity involving activation of calpains via the influx of Ca-ions [21]. Therefore, three quenched FRET probes were tested for cleavage by human calpain-1. This enzyme cleaved the quenched Ng39–51 and Ng31–40, but not the C-terminal probe Ng70–78 (Fig. 2).

**Fig. 3:**
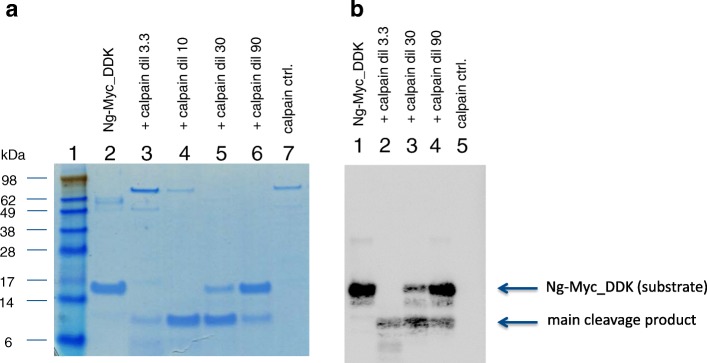
Cleavage of Ng1–78-Myc-DDK fusionprotein by calpain-1; **a** Coomassie stained SDS PAGE gel, Lane 1, size markers; lane 2, Ng-Myc-DDK substrate; lanes 3–6, digests with 3.3 to 90-fold diluted calpain stock solution; lane 7, calpain control; **b** Western blot of calpain-1 Ng1–78-Myc-DDK fusion protein digests by antibody NG22. Lane 1, Ng-Myc-DDK substrate; lanes 2–4, digests with 3.3, 30 and 90-fold diluted calpain stock solution; lane 5, calpain control. The positions of the substrate and of the main cleavage product are indicated

**Fig. 4:**
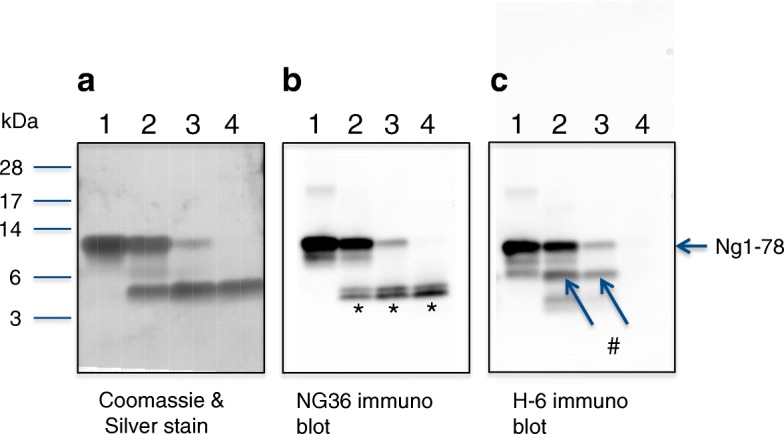
Detection of C-terminal and N-terminal fragments of Ng in digests by calpain. Digestion products were analyzed in reducing SDS PAGE gels (12%). Panel **a** Coomassie stain followed by silver stain; Panel **b** NG36 immunoblot; Panel c, H-6 immunoblot. Lanes 1, Ng control; lanes 2, 3 and 4, Ng digest with 90-, 30-, 10-fold diluted calpain-1, respectively. The location of the parent Ng1–78 band is indicated on the right near panel **c** with an arrow, those of the major ~ 5 kDa C-terminal fragments by an * in panel b, and those of the main N-terminal fragment near 6.5 kDa by a # in panel **c**. Size markers for panel a as indicated

**Fig. 5:**
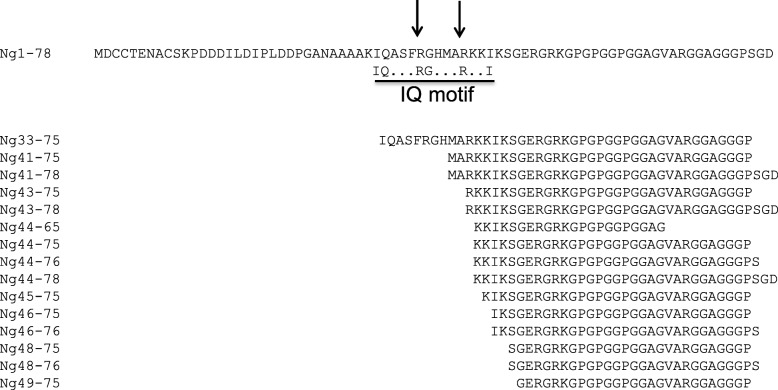
Alignment of identified endogenous Ng peptides in CSF with the full-length Ng amino acid sequence above. The identities of the peptides are given on the left, with the aa positions (N- and C-terminal) as indicated. Further, the line below the Ng sequence indicates the IQ domain with Ng’s conserved amino acid positions for the IQ motif above the line. The arrows indicate the calpain-1 cleavage sites between Ng37_38 and Ng42_43 which are generating the three most abundant fragments (Ng38–78, Ng43–78 and Ng1–37). Note that these cleavage sites are within the IQ domain

Calpain cleavage of Ng was further confirmed using recombinant full-length Ng substrate (Ng-Myc-DDK) in an in vitro cleavage assay with human calpain-1. As can be seen in Fig. 3a, lane 4, only one predominant band was observed, indicating one or more co-migrating cleavage products of about 8 kDa (apparent MW), which was also immunoreactive to the C-terminal region specific NG22 mAb (Fig. 3b).This places the cleavage site approximately in the same region as contained in the quenched FRET peptide Ng39–51. Further digestion with higher concentration of calpain-1 resulted in an additional weaker band at around 5–6 kDa (Fig. 3a, lane 3; seen also weakly in lane 4). Since the Myc-DDK tag on the fusion protein may have influenced the cleavage pattern on the Ng_Myc-DDK fusion protein, Ng was expressed in the SUMO expression system, which allows the complete removal of expression vector-derived amino acids from the expressed Ng. The SDS-PAGE-separated cleavage mix (Fig. 4a) showed the conversion of full-length Ng (approx. 12 kDa; Coomassie-silver stain, lane 1) to a major cleavage product of around 5 kDa (lanes 2–4) and a faintly stained band around 6.5 kDa (lanes 2, 3), thus indicating that the cleavage by Calpain-1 mainly occurs close to the center of the molecule. The N-terminal half of Ng is highly acidic and stains only weakly with Coomassie-silver stain; the band intensities therefore do not reflect quantities. The band at 5 kDa represented the C-terminal half of Ng, as seen by reactivity towards NG36 (a monoclonal antibody raised against Ng63–75) (Fig. 4b). This blot also revealed that the 5 kDa band was composed of at least two bands of similar size (difference estimated to about 700 Da), which appeared stable even at the highest calpain-1 concentration used (Fig. 4b, lane 4). These bands migrated and stained with Coomassie in about the same manner as synthetic Ng43–78 (data not shown); in contrast, the N-terminal half of Ng, as detected by the H-6 monoclonal antibody (raised against Ng1–50), generated several fragments of intermediate sizes and was further cleaved at higher calpain-1 concentrations (no band in lane 4 detected in Fig. 4c). The strongest intermediate band (around 6.5 kDa) co-migrated with synthetic Ng1–42 (data not shown). A more precise analysis of the cleavage positions (Fig. 5) was performed by mass spectrometry, which suggested that the three predominant fragments were Ng38–78, Ng43–78 and Ng1–37. Several, mostly N-terminal, shorter fragments were also detected in the digestion mix, indicating that the N-terminal half of Ng is readily cleaved by calpain-1, whereas the C-terminal half is more resistant to cleavage. These in vitro cleavage data may explain the occurrence of some, but not all, of the identified endogenous Ng peptides in CSF (Fig. 5). The identified peptides of Ng in CSF have N-terminal endings at aa 33, 41, 43, 44, 45, 46, 48 and 49 and most of them (eight out of fifteen peptides identified) end at aa 75 [6]. Thus, it appears that most of these fragments may have originated from cleavage at or around Ng42_43 and additional cleavage at Ng75_76.

### Identification of prolyl endopeptidase as the enzyme cleaving Ng near the C-terminus (aa 75_76)

As mentioned above, many of the endogenous Ng CSF peptides end C-terminally at aa 75. Calpain-1 did not cleave the quenched FRET probe Ng70–78 (Fig. 2). Thus, additional enzyme(s) may be responsible for the cleavage at position 75. Such C-terminally cleaved Ng peptides have been identified also in human brain tissue using MALDI TOF/TOF [6], indicating that brain tissue may be a good source for identifying the C-terminally cleaving enzyme.

**Fig. 6:**
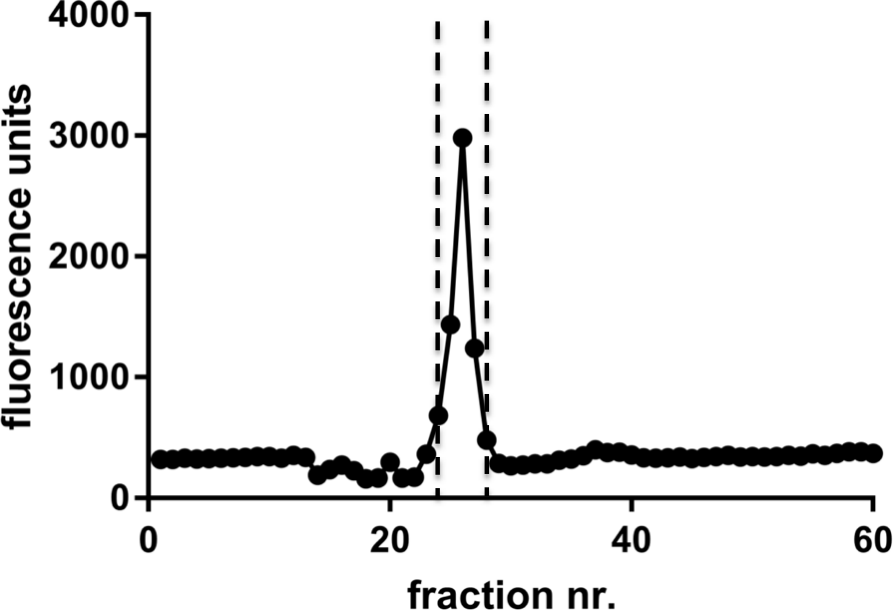
Activity profile of SEC column fractions (Superdex 200 size exclusion column). Active fractions in the Ng C-terminal cleaving assay are indicated by the two dashed vertical lines and corresponded to a protein size range of approximately 44–73 kDa

To increase the chance of detecting a C-terminal-cleaving activity on Ng in such a complex matrix, mouse brain extract was initially size-separated, and the fractions were further analyzed for C-terminal peptide cleaving activity (quenched FRET peptide representing Ng70–78). While the SEC column had the disadvantage of diluting the proteins during size separation, it was still preferred to an ion-exchange column for this initial experiment because of lower risk of losing activity on the column by strong adsorption. Active fractions were detected in the size range of about 44–73 kDa (Fig. 6).

**Fig. 7:**
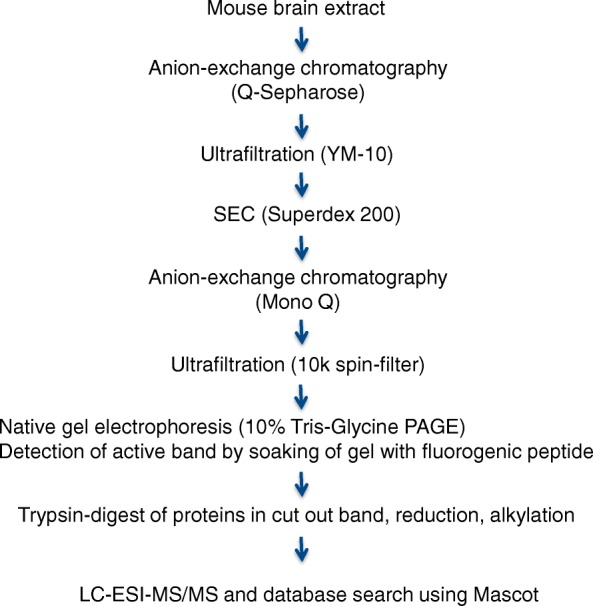
Strategy for enrichment of protease activity cleaving near the C-terminal end of Ng and identification of enzyme by mass spectrometry

**Fig. 8:**
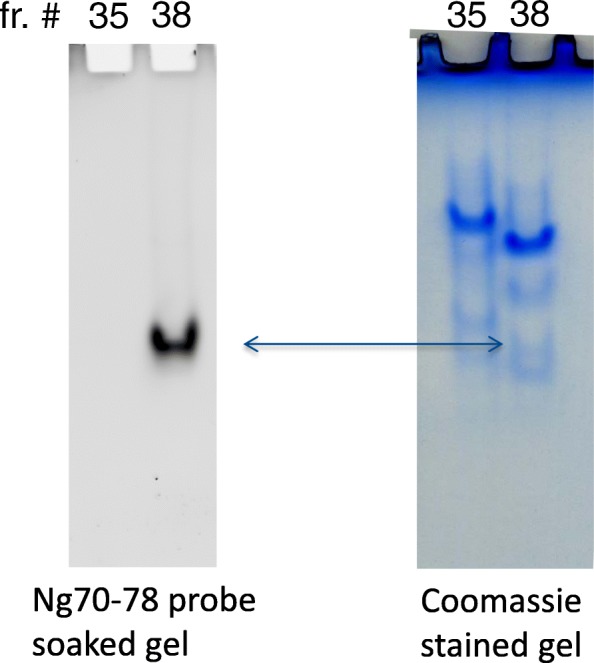
In situ localization of enzymatic activity in native PAGE separated proteins. Two fractions (fr. #35 and #38 with either no activity or with activity) were separated on a 10% Tris-glycine gel. The location of enzymatic activity was revealed thereafter by soaking the gel in fluorogenic quenched FRET peptide Ng70–78 (5-FAM_Ng70–78_QXL520). Note that only fraction #38 showed a band of fluorescence but not the nearby inactive fraction #35). The Coomassie-stained corresponding gel showed presence of proteins in both fractions. Arrow, location of enzymatic activity

**Fig. 9:**
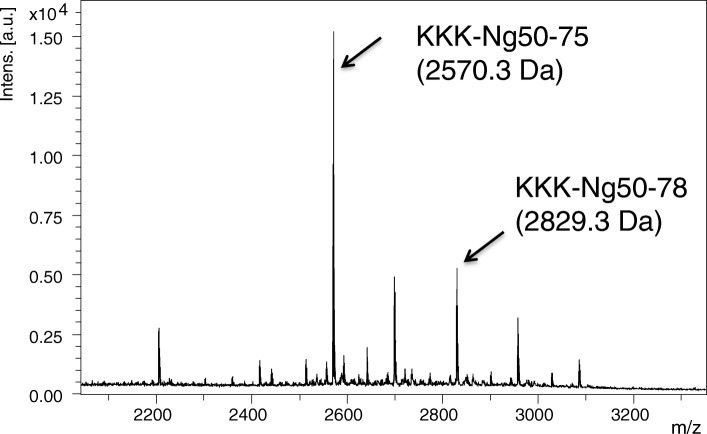
Active fractions during enzyme enrichment show cleaving activity at Ng75_76 (MALDI-TOF analysis). Synthetic KKK-Ng50–78 was incubated with an active fraction and the reaction mix separated by HPLC. MALDI-TOF analysis of one of the HPLC fractions showed the presence of an [M + H]^+^ peak at 2570.3 Da which is consistent with a cleavage at Ng75_76 which releases –SGD (mass difference of 259 Da relative to the substrate KKK-Ng50–78)

For a more detailed characterization of the active enzyme, a larger scale purification was required but the capacity of the size exclusion column was not sufficient. Therefore, a strategy involving an initial separation of mouse brain extract on an anion-exchange column, followed by ultrafiltration and buffer exchange to low salt buffer by SEC was chosen (see Fig. 7). Anion-exchange chromatography was then repeated on a high resolution anion-exchange resin. For the final identification of the C-terminal cleaving enzyme, fractions with high enzymatic activity from the anion-exchange column were further separated in a native polyacrylamide gel and bands containing enriched enzymatic activities localized by soaking the gel in the fluorogenic FRET Ng70–78 peptide and imaging of the gel in UV-light (Fig. 8). Fluorescent bands were cut out, reduced, alkylated, trypsinated and prepared for LC-MS/MS analysis. Using this strategy, sixty-two unique peptides of prolyl endopeptidase (Q9QUR6; PPCE mouse) with a sequence coverage of > 85% were identified (see Additional files 1 and 2). To confirm that active fractions indeed were able to cleave off the three last amino acids at the C-terminus of Ng (cleavage between Ng75 and Ng76), synthetic peptide KKK-Ng50–78 (human aa sequence) was incubated with the active fractions from the initial anion-exchange column (Q-Sepharose) and the resulting mix fractionated on a C18 reversed phase HPLC column. Figure 9 shows a MALDI -TOF spectrum of one of the fractions obtained. In the spectrum, a strong peak appears at 2570.3 Da, which is 259 Da less than the substrate KKK-Ng50–78 (2829.5 Da), thus indicating the presence of specific 75_76 cleaving activity. In addition, cross species activity to human PREP was confirmed by using either recombinant mouse or human PREP in a cleavage experiment on the human KKK-Ng50–78 peptide. Furthermore, an identical HPLC retention time of a newly formed elution peak with synthetic peptide KKK-Ng50–75 was observed for digestions of KKK-Ng50-78 with either mouse or human PREP enzyme. To extend the results to cleavage of larger Ng peptides, both either full length recombinant human Ng1–78 or a synthetic peptide of human Ng43–78 were digested in vitro with human PREP enzyme. Cleavage products Ng1–75 and Ng43–75, respectively, were identified by LC-MS/MS as minor products (approximately 1% MS signal area relative to remaining uncleaved Ng peptide).

## Discussion

Synaptic dysfunction and degeneration, which is key in AD pathophysiology [1, 22–24], will lead to secretion and/or release of synaptic proteins and their eventual increase in the CSF of AD patients. Indeed, increased Ng concentrations, as detected with sandwich ELISA and mass spectrometry, have shown to detect AD already at the mild cognitive impairment stage (MCI) of the disease [6] and may therefore be a useful clinical biomarker. Both full-length Ng and fragments of Ng have been detected in CSF by western blotting and mass spectrometry, respectively [5, 6]. The identified endogenous Ng peptides originated from the C-terminal half of the molecule [6].

This study focused on the proteolytic cleavage of Ng with the aim to identify the enzymes involved in its cleavages. Such cleavage may reduce the intracellular level of Ng, or change its location, and consequently attenuate the synaptic efficacy resulting in cognitive deficits. Ng knock out mice have difficulties with spatial memory and altered long-term potentiation (LTP) and long-term depression (LTD) [19]. On the other hand, increased levels of Ng have been reported to counteract the effects of Aβ on synaptic transmission and LTP [20]. It is most often unknown what physiological factor triggers the activation of proteases in the CNS that lead to the degradation of cellular components and neurodegenerative diseases. Information on the enzymatic cleavage of synaptic proteins such as Ng may shed some light not only into particular steps of the pathogenesis of AD, but also into physiologic functions such as LTP and LTD and offer options for pharmacological intervention. It may also lead to identification of specific fragments of Ng that reflect particular disease stages of AD and it may point to potentially different mechanisms of synaptic dysfunction suggested by the biomarker studies of Ng comparing various neurodegenerative diseases [8].

When comparing the sequences of 15 endogenous Ng peptides found in CSF [6], it became apparent that the sequence of most of the peptides started around aa 41–49, just at the C-terminal end of the IQ domain (aa 33–46) of Ng [25], and most of them lacked the very last three C-terminal amino acids (aa 76–78). Therefore, fluorogenic quenched FRET probes containing sequences of Ng of these cleavage regions were constructed. Three quenched probes were constructed, one containing Ng39–51 and another one containing Ng31–40, both for detecting the central Ng cleavage, and one with Ng70–78 for detecting C-terminal cleavage. Initially, the Ng39–51 central probe was used to screen various candidate enzymes reported to be activated during AD (such as BACE1, TACE-1, IDE, and calpain-1). Of those, cleaving activity was found with calpain-1 and this was confirmed by cleavage of recombinant Ng-Myc-DDK fusion protein, synthetic Ng1–78, and recombinant Ng (all with human sequence).

Increased activation of calpain-1 has been reported in brain samples of AD patients [26, 27] and several neuronal proteins are known substrates for calpain, e.g. αII and βII spectrin, PSD95, MAP-2, GAP-43, calcineurin, PKC, CaMKII, and NMDA receptors. While physiological activation may have roles in rearranging cytoskeletal proteins or during remodeling of spine structures as seen in LTP, pathological activation may lead to depletion of essential components of neuronal signaling. It is quite striking that the calpain-1 cleavage sites in Ng are either in or adjacent to the IQ domain. Calpain-1 activity thus generates fragments of Ng without an intact IQ domain, which thereby very likely lose their ability to bind to CaM. This in turn may lead to defective Ca^2+^/CaM-dependent protein kinase signaling with downstream effects on LTP (as seen in Ng knockout mice). The appearance of Ng fragments in CSF may therefore reflect such changes occurring in the brain. The sequence of Ng also contains a short sequence of basic amino acids (Ng43–47; RKKIK) which may act as a nuclear translocation sequence and could be responsible for binding activity to phosphatidic acid [28]. Upon cleavage of Ng by calpain-1 between amino acids 42_43, fragments beginning with this basic RKKIK sequence are formed and potentially exposed – which in contrast to the uncleaved Ng may have this RKKIK sequence covered by bound CaM or PA. This may lead to translocation events for the Ng fragments with consequences for cellular or nuclear signaling. Nevertheless, this hypothesis needs to be experimentally verified.

To embark on an unbiased screen to find enzymes responsible for the C-terminal cleavage of Ng, we probed mouse brain extract at various stages of chromatographic fractionation with the C-terminal fluorogenic quenched FRET probe Ng70–78. The anion exchange column fraction that was most enriched in enzymatic activity was further concentrated by ultrafiltration and finally separated by native PAGE. The fluorogenic FRET probe Ng70–78, soaked into the gel after the separation of the active fraction, revealed the localization of the enzymatic activity. The strongly fluorescing band was cut out and used for LC-ESI-MS/MS identification of the enzyme. Database searches identified mouse PREP (Uniprot ID Q9QUR6; EC:3.4.21.26) as the main hit, with 85% sequence coverage of the full-length protein by tryptic peptides. PREP belongs to the family of serine proteases, has a molecular weight of about 80 kDa and cleaves peptides C-terminally after proline [29]. Indeed, recombinant human and mouse PREP generated in vitro the C-terminally truncated fragment KKK-Ng50–75 from synthetic KKK-Ng50–78 substrate, however, other fragments derived by cleavage on other internal PREP cleavage sites on the substrate predominated.

PREP is a cytosolic neuronal protein that binds to microtubules and is also found in close association with cell membranes [30], bringing it potentially into vicinity of PA-bound Ng or other endogenous substrates. Additionally, PREP enzyme activity has been detected in CSF [31] using a sensitive HPLC method with fluorimetric detection. Inhibition of the PREP enzyme has been studied with regards to changes in Ins(1,4,5)P_3_ concentrations [32], improvements in memory function in rats and monkeys, and a phase I study has been reported in humans [33] . The known substrates of PREP are small neuropeptides peptides, < 30 amino acids (aa) long, such as substance P, thymosin β4. Longer peptides may potentially enter the active site of the enzyme, but only if they are of randomly disordered structure [34]. This raises the question whether full-length Ng can be a substrate because it is substantially larger (78 aa). Recombinant Ng1–78 and the calpain fragment of Ng, Ng43–78, generated only small amounts of Ng1–75 and Ng43–75, respectively, when incubated with fairly high concentrations of human PREP in vitro. Whether this low ability of PREP enzyme to cleave longer Ng peptides at Ng75_76 reflects a limitation of the in vitro assay or non-ideal properties the synthetic peptide used (e.g., due to cis/trans isomerism at proline positions, or lack of posttranslational modifications), or whether it is indeed due to a substrate size limitation of this enzyme, is currently not known. Activity of PREP requires conformational changes, and therefore interaction of a PREP enzyme-substrate complex with another cellular protein to form an enzymatically active tertiary complex may be needed [35]. A proteomic study in fractionated brain extracts revealed increased levels of α-synuclein and Ng peptides after in vitro incubation of brain extracts with added PREP enzyme [36]. Interestingly, the identified Ng48–56 peptide, SGERGRKGP, starts at the same N-terminal position (Ng48) as two of the endogenous Ng CSF peptides. Furthermore, another proteomic study using the PREP inhibitor S17092 in hypothalamus tissue extracts from mice, showed higher levels of Ng54–75 in vehicle treated animals vs. those treated with S17092 inhibitor [37]. The sequence of Ng also contains a number of other prolines after which PREP may also cleave. However, the cleavage sites were not mapped in detail, as it may depend on the substrate length and digestion conditions and it is difficult to know which applies to the intracellular conditions.

For the generation of Ng peptides, we currently favor a model which requires initial cleavage of Ng by enzymes active in neurons, such as calpain 1. This then delivers the shorter fragments for further cleavage by PREP and possibly, additional proteases, and eventually generating the fragments of Ng that can be detected in CSF. Consequently, patients enrolled in clinical trials for calpain- and/or PREP inhibitors may show decreased concentrations of Ng fragments in CSF. Future work will be needed to clarify the compartments in which Ng cleavages occur (intra-neuronal, in the interstitial fluid, or in CSF), the extent to which such cleavage occurs (fragment amounts relative to full-length Ng) and its timing in relation to AD pathology. This may give insights about the role of Ng cleavage for the Ng reduction seen in AD brain, and provide additional enzyme-specific fragments of Ng as potential biomarkers for specific stages of AD.

## Conclusions

Calpain-1- and prolyl endopeptidase were identified as enzymes generating the pattern of C-terminal fragments of Ng detectable in CSF. Calpain-1 cleaves Ng in the IQ domain, while prolyl endopeptidase cleaves after prolines, resulting in C-terminal Ng peptides ending at Ng75 which are predominantly found in CSF.

## Additional files

Additional file 1: Top 30 hits from Mascot search in Swiss-Prot (mouse) using tryptic peptides from excised band of C-terminal Ng cleaving activity. (DOCX 37 kb) (XLSX 10 kb)

Additional file 2: Mascot search engine data for peptides identified in tryptic digest of excised protein band with cleavage activity on FRET peptide Ng70–78. (DOCX 37 kb)

## Abbreviations

Aa: Amino acid
AD: Alzheimer’s disease
CaM: Calmodulin
CNS: Central nervous system
CSF: Cerebrospinal fluid
Da: Dalton
ESI: Electrospray ionization
FRET: Fluorescence resonance energy transfer
LC: Liquid chromatography
LTD: Long term depression
LTP: Long term potentiation
MS: Mass spectrometry
MW: Molecular weight
Ng: Neurogranin
PA: Phosphatidic acid
PBS: Phosphate buffered saline
PREP: Prolyl endopeptidase
SEC: Size-exclusion chromatography
SUMO: Small ubiquitin-like modifier
