# Supplementary material for: Alzheimer-associated cerebrospinal fluid fragments of neurogranin are generated by Calpain-1 and prolyl endopeptidase
Source: Mol Neurodegener. 2018 Aug 29;13:47. doi: 10.1186/s13024-018-0279-z (PMC6116393; doi:10.1186/s13024-018-0279-z)
Supplement: Supplementary file 2 — Mascot search engine data for peptides identified in tryptic digest of excised protein band with cleavage activity on FRET peptide Ng70–78. (DOCX 37 kb) [file 13024_2018_279_MOESM2_ESM.docx]

Additional file 2

Matched peptides underlined

(in sequence of UniProt Q9QUR6)

001 MLSFQYPDVY RDETSVQEYH GHKICDPYSW LEDPDSEQTK AFVEAQNKIT

051 VPFLEQCPIR GLYKERMTEL YDYPKYSCHF KKGKRYFYFY NTGLQNQRVL

101 YVQDSLEGEA RVFLDPNTLS DDGTVALRGY AFSEDGEYFA YGLSASGSDW

151 VTIKFMKVDG AKELPDVLER VKFTCMAWTH DGKGMFYNSY PQQDGKSDGT

201 ETSTNLHQKL CYHVLGTDQS EDILCAEFPD EPKWMGGAEL SDDGRYVLLS

251 IWEGCDPVNR LWYCDLQQEP NGITGILKWV KLIDNFEGEY DYVTNEGTVF

301 TFKTNRNSPN YRLINIDFTD PDESKWKVLV PEHEKDVLEW VACVRSNFLV

351 LCYLHDVKNI LQLHDLTTGA LLKTFPLDVG SVVGYSGRKK DSEIFYQFTS

401 FLSPGVIYHC DLTKEELEPM VFREVTVKGI DAADYQTIQI FYPSKDGTKI

451 PMFIVHKKGI KLDGSHPAFL YGYGGFNISI TPNYSVSRLI FVRHMGGVLA

501 VANIRGGGEY GETWHKGGIL ANKQNCFDDF QCAAEYLIKE GYTSPKRLTI

551 NGGSNGGLLV AACANQRPDL FGCVIAQVGV MDMLKFHKFT IGHAWTTDYG

601 CSDTKQHFEW LLKYSPLHNV KLPEADDIQY PSMLLLTADH DDRVVPLHSL

651 KFIATLQYIV GRSRKQSNPL LIHVDTKAGH GAGKPTAKVI EEVSDMFAFI

701 ARCLNIEWIQ

| Positions | M [Da] | Δm [ppm] | Missed cleav. | Mascot score | Expect value | Sequence |
| --- | --- | --- | --- | --- | --- | --- |
| 024-040 | 2081.889 | 0.69 | 0 | 91 | 8.80E-10 | K.ICDPYSWLEDPDSEQTK.A |
| 041-048 | 905.4607 | -0.39 | 0 | 53 | 1.00E-05 | K.AFVEAQNK.I |
| 049-060 | 1471.786 | 0.77 | 0 | 81 | 2.80E-08 | K.ITVPFLEQCPIR.G |
| 049-060 | 1471.786 | 1.76 | 0 | 81 | 4.30E-08 | K.ITVPFLEQCPIR.G |
| 049-060 | 1471.786 | 3.33 | 0 | 82 | 2.30E-08 | K.ITVPFLEQCPIR.G |
| 065-075 | 1459.665 | -0.81 | 1 | 34 | 1.50E-03 | K.ERMTELYDYPK.Y + Ox (M) |
| 067-075 | 1158.527 | 0.12 | 0 | 64 | 1.30E-06 | R.MTELYDYPK.Y |
| 067-075 | 1174.522 | 0.39 | 0 | 54 | 1.00E-05 | R.MTELYDYPK.Y + Ox (M) |
| 085-098 | 1868.896 | 1.14 | 1 | 60 | 4.70E-06 | K.RYFYFYNTGLQNQR.V |
| 086-098 | 1712.795 | 0.76 | 0 | 88 | 6.30E-09 | R.YFYFYNTGLQNQR.V |
| 099-111 | 1477.741 | 1.85 | 0 | 86 | 8.20E-09 | R.VLYVQDSLEGEAR.V |
| 099-128 | 3291.662 | -0.13 | 1 | 29 | 2.60E-03 | R.VLYVQDSLEGEARVFLDPNTLSDDGTVALR.G |
| 112-128 | 1831.932 | 0.53 | 0 | 94 | 1.80E-09 | R.VFLDPNTLSDDGTVALR.G |
| 129-154 | 2819.26 | 0.05 | 0 | 77 | 3.90E-08 | R.GYAFSEDGEYFAYGLSASGSDWVTIK.F |
| 155-162 | 910.4582 | 0.12 | 1 | 33 | 7.60E-04 | K.FMKVDGAK.E + Ox (M) |
| 158-170 | 1439.762 | -0.17 | 1 | 69 | 3.50E-07 | K.VDGAKELPDVLER.V |
| 163-170 | 969.5131 | 0.26 | 0 | 46 | 9.60E-05 | K.ELPDVLER.V |
| 171-183 | 1579.728 | 0.56 | 1 | 49 | 4.80E-05 | R.VKFTCMAWTHDGK.G |
| 171-183 | 1595.723 | 8.64 | 1 | 10 | 4.00E-01 | R.VKFTCMAWTHDGK.G + Ox (M) |
| 173-183 | 1352.564 | 0.72 | 0 | 65 | 4.20E-07 | K.FTCMAWTHDGK.G |
| 173-183 | 1368.559 | 1.21 | 0 | 78 | 2.10E-08 | K.FTCMAWTHDGK.G + Ox (M) |
| 184-196 | 1533.656 | 0.90 | 0 | 69 | 1.30E-07 | K.GMFYNSYPQQDGK.S |
| 184-196 | 1549.651 | 1.01 | 0 | 69 | 1.40E-07 | K.GMFYNSYPQQDGK.S + Ox (M) |
| 184-209 | 2932.293 | 0.39 | 1 | 46 | 2.70E-05 | K.GMFYNSYPQQDGKSDGTETSTNLHQK.L |
| 184-209 | 2948.288 | 0.04 | 1 | 44 | 3.70E-05 | K.GMFYNSYPQQDGKSDGTETSTNLHQK.L + Ox (M) |
| 210-233 | 2835.273 | 1.38 | 0 | 66 | 4.90E-07 | K.LCYHVLGTDQSEDILCAEFPDEPK.W |
| 210-245 | 4125.803 | -0.39 | 1 | 33 | 4.50E-04 | K.LCYHVLGTDQSEDILCAEFPDEPKWMGGAELSDDGR.Y + Ox (M) |
| 234-245 | 1292.546 | 0.73 | 0 | 71 | 1.10E-07 | K.WMGGAELSDDGR.Y |
| 234-245 | 1308.541 | 1.06 | 0 | 83 | 6.50E-09 | K.WMGGAELSDDGR.Y + Ox (M) |
| 246-260 | 1819.893 | 0.99 | 0 | 76 | 6.90E-08 | R.YVLLSIWEGCDPVNR.L |
| 261-278 | 2147.072 | 0.12 | 0 | 89 | 4.40E-09 | R.LWYCDLQQEPNGITGILK.W |
| 282-303 | 2600.196 | 0.29 | 0 | 116 | 8.90E-12 | K.LIDNFEGEYDYVTNEGTVFTFK.T |
| 282-306 | 2971.388 | -0.08 | 1 | 50 | 2.50E-05 | K.LIDNFEGEYDYVTNEGTVFTFKTNR.N |
| 313-325 | 1505.725 | 1.05 | 0 | 66 | 7.20E-07 | R.LINIDFTDPDESK.W |
| 313-327 | 1819.899 | 0.83 | 1 | 81 | 2.70E-08 | R.LINIDFTDPDESKWK.V |
| 328-335 | 949.5233 | -0.17 | 0 | 36 | 1.10E-03 | K.VLVPEHEK.D |
| 328-345 | 2177.13 | -0.15 | 1 | 64 | 1.50E-06 | K.VLVPEHEKDVLEWVACVR.S |
| 336-345 | 1245.618 | 0.92 | 0 | 56 | 6.10E-06 | K.DVLEWVACVR.S |
| 346-358 | 1606.818 | 0.97 | 0 | 80 | 3.10E-08 | R.SNFLVLCYLHDVK.N |
| 359-373 | 1648.951 | 1.60 | 0 | 83 | 6.10E-09 | K.NILQLHDLTTGALLK.T |
| 374-388 | 1552.789 | 1.24 | 0 | 79 | 3.70E-08 | K.TFPLDVGSVVGYSGR.K |
| 390-414 | 2994.447 | 0.90 | 1 | 51 | 2.60E-05 | K.KDSEIFYQFTSFLSPGVIYHCDLTK.E |
| 391-423 | 3996.895 | 1.84 | 1 | 3 | 9.40E-01 | K.DSEIFYQFTSFLSPGVIYHCDLTKEELEPMVFR.E |
| 415-423 | 1148.554 | 0.95 | 0 | 62 | 1.90E-06 | K.EELEPMVFR.E |
| 415-423 | 1164.549 | 0.37 | 0 | 61 | 1.70E-06 | K.EELEPMVFR.E + Ox (M) |
| 424-445 | 2485.274 | 1.97 | 1 | 61 | 2.50E-06 | R.EVTVKGIDAADYQTIQIFYPSK.D |
| 429-445 | 1928.952 | 0.26 | 0 | 146 | 1.60E-14 | K.GIDAADYQTIQIFYPSK.D |
| 429-449 | 2330.143 | -1.10 | 1 | 56 | 1.20E-05 | K.GIDAADYQTIQIFYPSKDGTK.I |
| 446-457 | 1384.754 | -0.15 | 1 | 45 | 1.40E-04 | K.DGTKIPMFIVHK.K |
| 446-457 | 1400.749 | 0.43 | 1 | 42 | 1.10E-04 | K.DGTKIPMFIVHK.K + Ox (M) |
| 450-457 | 983.5626 | -1.41 | 0 | 42 | 1.30E-04 | K.IPMFIVHK.K |
| 450-457 | 999.5576 | 0.21 | 0 | 34 | 2.10E-03 | K.IPMFIVHK.K + Ox (M) |
| 450-458 | 1111.658 | 0.08 | 1 | 19 | 2.20E-02 | K.IPMFIVHKK.G |
| 450-458 | 1127.653 | 0.65 | 1 | 21 | 2.80E-02 | K.IPMFIVHKK.G + Ox (M) |
| 462-488 | 2931.419 | 0.79 | 0 | 67 | 8.20E-07 | K.LDGSHPAFLYGYGGFNISITPNYSVSR.L |
| 489-505 | 1881.077 | 0.86 | 1 | 51 | 9.40E-06 | R.LIFVRHMGGVLAVANIR.G + Ox (M) |
| 494-505 | 1236.676 | -0.08 | 0 | 74 | 1.40E-07 | R.HMGGVLAVANIR.G |
| 494-505 | 1252.671 | -0.31 | 0 | 80 | 3.40E-08 | R.HMGGVLAVANIR.G + Ox (M) |
| 494-505 | 1252.671 | 1.25 | 0 | 73 | 1.30E-07 | R.HMGGVLAVANIR.G + Ox (M) |
| 506-516 | 1219.526 | 3.35 | 0 | 60 | 2.00E-06 | R.GGGEYGETWHK.G |
| 506-523 | 1872.912 | -0.01 | 1 | 22 | 8.80E-03 | R.GGGEYGETWHKGGILANK.Q |
| 517-539 | 2674.252 | -1.75 | 1 | 62 | 1.80E-06 | K.GGILANKQNCFDDFQCAAEYLIK.E |
| 524-539 | 2020.866 | 1.41 | 0 | 101 | 9.50E-11 | K.QNCFDDFQCAAEYLIK.E |
| 524-546 | 2783.221 | 0.38 | 1 | 29 | 1.30E-03 | K.QNCFDDFQCAAEYLIKEGYTSPK.R |
| 589-605 | 1958.847 | -0.01 | 0 | 83 | 5.30E-09 | K.FTIGHAWTTDYGCSDTK.Q |
| 606-613 | 1099.582 | 0.72 | 0 | 47 | 1.40E-04 | K.QHFEWLLK.Y |
| 614-621 | 956.508 | 0.21 | 0 | 34 | 1.80E-03 | K.YSPLHNVK.L |
| 622-643 | 2527.19 | -0.97 | 0 | 108 | 5.10E-11 | K.LPEADDIQYPSMLLLTADHDDR.V |
| 622-643 | 2543.185 | 0.07 | 0 | 99 | 4.00E-10 | K.LPEADDIQYPSMLLLTADHDDR.V + Ox (M) |
| 622-651 | 3400.734 | 0.62 | 1 | 38 | 3.70E-04 | K.LPEADDIQYPSMLLLTADHDDRVVPLHSLK.F |
| 622-651 | 3416.729 | 0.90 | 1 | 34 | 8.60E-04 | K.LPEADDIQYPSMLLLTADHDDRVVPLHSLK.F + Ox (M) |
| 644-651 | 891.5542 | 0.88 | 0 | 35 | 3.50E-04 | R.VVPLHSLK.F |
| 644-662 | 2153.273 | 1.45 | 1 | 86 | 2.40E-09 | R.VVPLHSLKFIATLQYIVGR.S |
| 652-662 | 1279.729 | 0.95 | 0 | 80 | 2.20E-08 | K.FIATLQYIVGR.S |
| 665-677 | 1491.841 | 1.31 | 1 | 81 | 2.20E-08 | R.KQSNPLLIHVDTK.A |
| 666-677 | 1363.746 | 0.98 | 0 | 73 | 2.50E-07 | K.QSNPLLIHVDTK.A |
| 666-688 | 2339.271 | -0.13 | 1 | 14 | 9.70E-02 | K.QSNPLLIHVDTKAGHGAGKPTAK.V |
| 689-702 | 1625.812 | 1.30 | 0 | 103 | 2.40E-10 | K.VIEEVSDMFAFIAR.C |
| 689-702 | 1641.807 | 1.48 | 0 | 88 | 5.40E-09 | K.VIEEVSDMFAFIAR.C + Ox (M) |
| 703-710 | 1074.517 | 0.14 | 0 | 40 | 1.70E-04 | R.CLNIEWIQ.- |
